# Supplementary material for: Nutrition-Related Information on Alcoholic Beverages in Victoria, Australia, 2021
Source: Int J Environ Res Public Health. 2022 Apr 11;19(8):4609. doi: 10.3390/ijerph19084609 (PMC9030476; doi:10.3390/ijerph19084609)
Supplement: Supplementary file 1 [file ijerph-19-04609-s001.zip › ijerph-1620284-SI/Supplementary File S1.pdf]

Supplementary File S1

Beverage Name	Manufacturer	Category	Sub-Category	Content Information Included (e.g., energy, carbohydrate)	Claim Included	Format (e.g., NIP, FOP)	Other Notes

Figure S1. Self-developed audit tool used to collect data from labels of alcohol product available for purchase in Victoria, Australia in July 2021.
