# Supplementary material for: Nutrition-Related Information on Alcoholic Beverages in Victoria, Australia, 2021
Source: Int J Environ Res Public Health. 2022 Apr 11;19(8):4609. doi: 10.3390/ijerph19084609 (PMC9030476; doi:10.3390/ijerph19084609)
Supplement: Supplementary file 1 [file ijerph-19-04609-s001.zip › ijerph-1620284-SI/Supplementary File S2.pdf]

Supplementary File S2

	Beer	Wine	Spirits	RTDs	Ciders
**Beer**		43.05<br>< 0.001	43.05<br>< 0.001	28.37<br>< 0.001	14.81<br>< 0.001
**Wine**	43.05<br>< 0.001		0.00<br>1.00	120.46<br>< 0.001	3.557<br>0.06
**Spirits**	43.05<br>< 0.001	0.00<br>1.00		120.46<br>< 0.001	3.557<br>0.06
**RTDs**	28.37<br>< 0.001	120.46<br>< 0.001	120.46<br>< 0.001		60.66<br>< 0.001
**Ciders**	14.81<br>< 0.001	3.557<br>0.06	3.557<br>0.06	60.66<br>< 0.001	

Figure S2. Results of Chi-squared test to test for differences in prevalence of nutrient/energy level presentation between alcohol beverage categories available for purchase in Victoria, Australia in July 2021.

	Beer	Wine	Spirits	RTDs	Ciders
**Beer**		11.30<br>< 0.001	26.09<br>< 0.001	14.93<br>< 0.001	2.75<br>0.10
**Wine**	11.30<br>< 0.001		5.61<br>0.02	46.10<br>< 0.001	1.55<br>0.21
**Spirits**	26.09<br>< 0.001	5.61<br>0.02		67.04<br>< 0.001	11.61<br>< 0.001
**RTDs**	14.93<br>< 0.001	46.10<br>< 0.001	67.04<br>< 0.001		20.85<br>< 0.001
**Ciders**	2.75<br>0.10	1.55<br>0.21	11.61<br>< 0.001	20.85<br>< 0.001	

Figure S3. Results of Chi-squared test to test for differences in prevalence of nutrition content claim presentation between alcohol beverage categories available for purchase in Victoria, Australia in July 2021.
